# Polypyrimidine-Tract-Binding Protein Isoforms Differentially Regulate the Hepatitis C Virus Internal Ribosome Entry Site

**DOI:** 10.3390/v15010008

**Published:** 2022-12-20

**Authors:** Jenniffer Angulo, C. Joaquín Cáceres, Nataly Contreras, Leandro Fernández-García, Nathalie Chamond, Melissa Ameur, Bruno Sargueil, Marcelo López-Lastra

**Affiliations:** 1 Laboratorio de Virología Molecular, Centro de Investigaciones Médicas, Instituto Milenio de Inmunología e Inmunoterapia, Departamento de Enfermedades Infecciosas e Inmunología Pediátrica, Escuela de Medicina, Pontificia Universidad Católica de Chile, Santiago 8330024, Chile; 2 Facultad de Odontología, Universidad Finis Terrae, Santiago 7501015, Chile; 3 Department of Population Health, College of Veterinary Medicine, University of Georgia, Athens, GA 30602, USA; 4 Facultad de Medicina Veterinaria y Agronomía, Universidad de Las Américas, Santiago 7500975, Chile; 5 Centre National de la Recherche Scientifique, Unité Mixte de Recherche 8038, Laboratoire CiTCoM, Université Paris Cité, 75006 Paris, France

**Keywords:** HCV, IRES, PTB, ITAF

## Abstract

Translation initiation of the hepatitis C virus (HCV) mRNA depends on an internal ribosome entry site (IRES) that encompasses most of the 5′UTR and includes nucleotides of the core coding region. This study shows that the polypyrimidine-tract-binding protein (PTB), an RNA-binding protein with four RNA recognition motifs (RRMs), binds to the HCV 5′UTR, stimulating its IRES activity. There are three isoforms of PTB: PTB1, PTB2, and PTB4. Our results show that PTB1 and PTB4, but not PTB2, stimulate HCV IRES activity in HuH-7 and HEK293T cells. In HuH-7 cells, PTB1 promotes HCV IRES-mediated initiation more strongly than PTB4. Mutations in PTB1, PTB4, RRM1/RRM2, or RRM3/RRM4, which disrupt the RRM’s ability to bind RNA, abrogated the protein’s capacity to stimulate HCV IRES activity in HuH-7 cells. In HEK293T cells, PTB1 and PTB4 stimulate HCV IRES activity to similar levels. In HEK293T cells, mutations in RRM1/RRM2 did not impact PTB1′s ability to promote HCV IRES activity; and mutations in PTB1 RRM3/RRM4 domains reduced, but did not abolish, the protein’s capacity to stimulate HCV IRES activity. In HEK293T cells, mutations in PTB4 RRM1/RRM2 abrogated the protein’s ability to promote HCV IRES activity, and mutations in RRM3/RRM4 have no impact on PTB4 ability to enhance HCV IRES activity. Therefore, PTB1 and PTB4 differentially stimulate the IRES activity in a cell type-specific manner. We conclude that PTB1 and PTB4, but not PTB2, act as IRES transacting factors of the HCV IRES.

## 1. Introduction

The hepatitis C virus (HCV) is a hepatotropic, positive-sense, enveloped, single-stranded RNA virus; a member of the *Hepacivirus* genus of the *Flaviviridae* family. The HCV mRNA of ~9.6 kb has an open reading frame (ORF) flanked by highly conserved 5′ and 3′ untranslated regions (UTRs), and it encodes for a polyprotein of approximately 3000 amino acids (aa). Translation initiation of the HCV mRNA exclusively depends on an internal ribosome entry site (IRES) that encompasses most of the 5′UTR and includes nucleotides of the Core coding region [1,2,3]. The HCV IRES directly recruits the 40S ribosomal subunit, positioning the AUG initiation codon into the ribosomal P-site without requiring any eukaryotic initiation factor (eIF) [4,5]. Host RNA binding proteins, IRES-transacting factors (ITAFs), and the ribosomal proteins RACK1 and eS25 influence HCV IRES activity [6,7,8,9,10,11,12,13,14,15,16,17]. Among the known HCV IRES ITAFs, the La autoantigen [6,7], heterogeneous nuclear ribonucleoprotein L (hnRNPL) [8], hnRNPD [9], Hu-antigen R (HuR) [10], PSMA7 [10,11], NS1-associated protein 1 (NSAP1) [12,13], Lsm1–7 complexes [14], and nucleolin [15] promote HCV IRES activity. In contrast, Gemin5 and RNA binding motif protein 24 (RBM24) down-regulate HCV IRES-mediated translation initiation [16,17]. Polypyrimidine-tract binding protein (PTB or PTBP1) has also been proposed to be an ITAF for the HCV IRES [18,19,20,21,22]. However, data are conflicting, as one report suggests that PTB inhibits HCV IRES-mediated translation [23], whereas others indicate that PTB has little or no impact on HCV IRES activity [21,24,25,26].

PTB is a nucleo-cytoplasmic shuttling protein with known biological functions in the nucleus and the cytoplasm [27,28,29]. As a result of alternative splicing, three isoforms of PTB exist: PTB1 (531 amino acids (aa)), PTB2 (550 aa), and PTB4 (557 aa) [29,30,31,32]. PTB isoforms are differentially expressed in different cell types [33,34,35]. PTBs have four RNA recognition motifs (RRM1–4) [36,37,38,39,40], tethered together by conserved linker domains, which bind with high affinity to RNAs containing pyrimidine-rich tracts with the core sequence UCUU or UCUUC [33,37,41,42]. However, each RRM has a slightly different consensus RNA-binding sequence [39,43]. RRM1 and RRM2 are independent domains connected by flexible linkers, whereas RRM3 and RRM4 have a fixed relative orientation [39]. Compared to PTB1, PTB2 and PTB4 contain 19 and 26 additional amino acids between RRM2 and RRM3, respectively [29,32]. Thus, PTB isoforms differ only in the alternatively spliced linker sequences between RRM2 and RRM3. Interestingly, despite their similarity, PTB isoforms exhibit different functions in mRNA splicing and regulating the activity of IRESs [32,44,45,46].

In this study, we use a reporter plasmid encoding for a bicistronic mRNA harboring the HCV 5′UTR to evaluate the impacts of PTB isoforms on HCV IRES activity in two human cell lines, HuH-7 and HEK293T. The results show that PTB2 does not influence HCV IRES activity, and PTB1 and PTB4 differentially promote HCV IRES activity in a cell-type-dependent fashion.

## 2. Material and Methods

**Plasmids.** The dl HCV IRES and dl ∆EMCV plasmid were previously described [47,48]. The PTB-tagged expressing plasmids (pcDNA4/HisMax-PTB; pcDNA4/HisMax-PTB2; pcDNA4/HisMax-PTB4, pCINeo-PTB2-FLAG; pCINeo-PTB4-FLAG; pcDNA4/HisMax-PTB1m1.2; pcDNA4/HisMax-PTB1m3.4; pCINeo-PTB4m1.2-FLAG; pCINeo-PTB4m3.4-FLAG) were described in [46,49]. The promoterless vectors ∆SV40 dl ∆EMCV and ∆SV40 dl HCV-IRES were constructed as described in [46,50]. The commercial plasmid, pSP64-Poly(A) (#P1241, Promega Corporation, Madison, WI, USA), was used to standardize the amount of DNA transfected in every condition. The same amount of total DNA was used in every transfection assay. Plasmid pcDNA3.1-LacZ, which expresses β-galactosidase described in (65, 69), was used to control the transfection efficiency. The pNL4.3 DNA (HIV-1 vector; GenBank: AF324493) was kindly provided by the NIH AIDS Reference and Reagent Program. The pQE9-PTB1 plasmid used to generate recombinant PTB1 protein for the filter binding experiments was a generous gift of P. Kafasla (Department of Biochemistry, University of Cambridge, UK).

**Viral mRNA 5′UTRs.** The DNA sequence corresponding to the 5′UTR of the HCV was amplified from the dl HCV IRES plasmid [47] using FV30 [51] as a 5′sense primer starting with a T7 promoter, followed by the following 16 nucleotides, 5′CCATATGTAATACGACTCACTATAGGGTTGGGGGCGACACTCC 3′, and with FLuc2 as the 3′antisense primer: 5′-TGGAAGATGGAACCGCTGGAGAG-3′. The amplification yields 477 bp long DNA fragments that transcribe into a 452 nt-long RNA starting nucleotide 13 of the HCV 5′UTR and ending within the luciferase coding region. The DNA sequence corresponding to the 5′UTR of EMCV was amplified by PCR using a 5′ oligonucleotide starting with the T7 promoter sequence, followed by 22nt encoding EMCV 5′UTR_265–287_ (5′-TAATACGACTCACTATAGCCCCTCTCCCTCCCCCTAAACG-3′) and the 5′-AACATTCATTTGTTTACATCTG-3′ oligonucleotide. The DNA sequence corresponding to the 5′UTR of the HIV-1 RNA was amplified by PCR from the pNL4.3 plasmid using a 5′ oligonucleotide starting with the T7 promoter sequence followed by the first 25 nt of HIV-1 5′UTR (5′-TAATACGACTCACTATAGGGGTCTCTCTGGTTAGACCAGATCT-3′) and the 5′-CTCCTTCTAGCCTCCGCTAGTC-3′oligonucleotide. All RNAs were directly transcribed using the T7 RNA polymerase from PCR products containing the T7 polymerase promoter sequence and purified as previously described [52,53]. Radiolabeled RNAs were transcribed as above in the presence of α-^32^P-UTP (3000 mCi/mmol) and purified by size exclusion chromatography.

**PTB1 purification and Filter Binding assays.** Plasmid pQE9-PTB1 was transformed into BL21 (lambda DE3) *Escherichia coli*, and PTB1 was produced, purified, and stored as detailed in [46]. Radiolabeled RNA was denatured in water for 2 min at 80 °C and then cooled to room temperature in binding buffer (20 mM Tris pH 7.6, 100 mM KOAc, 2 mM DTT, 2.5 mM MgCl_2_, and 0.25 mM spermidine). Serial dilution of PTB1 were prepared extemporaneously, added to a 10 µL reaction containing 5 pmoles of RNA, and incubated at 30 °C for 20 min. Filter binding was conducted using two filters, using an upper nitrocellulose filter (#WHA10401114, Sigma-Aldrich, St. Louis, MO, USA) and a lower charged nylon filter (#Z290807, Sigma-Aldrich), as previously described [54]. The filters were presoaked in 1X binding buffer and assembled in a dot blot apparatus, and the reactions were applied and directly vacuum-filtered. The filters were then rinsed, removed, and dried, and radioactivity was quantified using a Storm phosphorImager (GE Healthcare Life Science, Logan, UT, USA).

**Cell culture.** The Human hepatocellular carcinoma cell line HuH-7 [47] and the human embryonic kidney cells (HEK293T, ATCC, CRL-11268) were grown in Dulbecco’s modified eagle’s medium (DMEM; #SH30022, HyClone, GE Healthcare Life Sciences) containing 10% fetal bovine serum (#SH30910, Hyclone), 1% penicillin-streptomycin (1000 U/mL) (#SV30010, Hyclone), and 1% amphotericin B (25 mg/mL) (#SV30078.01, Hyclone), at 37 °C in a 5% CO_2_ atmosphere. In the case of HuH-7 cells, cultures were supplemented with nonessential amino acids (#11140-050; Gibco BRL, Life Technologies Corporation, Carlsbad, CA, USA).

**DNA transfection.** HuH-7 cells (7.5 × 10^4^ cells per well) or HEK293T cells (5.5 × 10^4^ cells per well) were seeded in 24-well culture plates. At 60% of confluency, cells were cotransfected with 100 ng of dl plasmid (dl HCV-IRES), together with increasing amounts of plasmid pcDNA4/HisMax-PTB1, pcDNA4/HisMax-PTB2, and pcDNA4/HisMax-PTB4) and pcDNA3.1-LacZ (25 ng) using polyethyleneimine (PEI; #23966 Gibco BRL, Life Technologies Corporation, Carlsbad, CA, USA). Plasmid pcDNA3.1-LacZ was used as a control for transfection efficiency. Plasmid pSP64-Poly(A) was used as filling DNA to keep the total amount of transfected DNA equal in all experiments. For experiments using the mutant PTBs, the dl HCV-IRES plasmid (100 ng) was cotransfected with 500, 750, and 1000 ng of the PTBs expression vectors plus pcDNA3-1-LacZ. For the IRES activity and cryptic promoter experiments, cells were seeded well in a 24-well culture plate and transfected at 60–70% confluency with 200 ng of bicistronic construct plus pcDNA3.1-LacZ using PEI. When combinations of PTB isoforms were used, Huh7 cells were transfected with the dl HCV IRES (100 ng) and pcDNA3.1-LacZ DNA (25 ng), 500 ng of plasmids expressing PTB1 or PTB4, and increasing concentrations (100–700 ng) of plasmids expressing a different PTB isoform as indicated in the figure legend. The total amount of DNA was constant in all conditions, as pSP64-Poly(A) was used as filler DNA. Twenty-four after transfection, the culture medium was removed, and the cells were harvested using the Passive Lysis buffer supplied with the DLR^TM^ Assay System (#E1910, Promega Corporation) according to the manufacturer’s protocols. The transfection efficiency of each sample was estimated by measuring β-galactosidase activity using the Beta-Glo^TM^ Assay System #E4720, Promega Corporation) according to the manufacturer’s instructions on a Sirius Single Tube Luminometer (Berthold Detection Systems GmbH, Pforzheim, Germany).

**Cytotoxic Assay.** HuH-7 cells were seeded at 2.5 × 10^4^ cells per well in 96-well culture plates. At 60% of confluency, cells were cotransfected with the dl plasmid (dl HCV-IRES) together with increasing amounts of plasmids pcDNA4/HisMax-PTB1, pcDNA4/HisMax-PTB2, and pcDNA4/HisMax-PTB4 using PEI. The total amount of transfected DNA was equal in all experiments, as pSP64-Poly(A) was used as filler DNA. Cells treated with 10% of DMSO were used as a positive control for cell death. Transfection reagents NaCl and NaCl plus PEI, without DNA, were incorporated into the experiment as additional controls. After 20 h, 20 µL of CellTiter 96^®^ AQueous One Solution Reagent (#E4720, Promega Corporation) was added to each well, and plates were incubated for 4 h at 37 °C in a humidified, 5% CO_2_ atmosphere. The absorbance was measured at 490 nm using a 96-well plate reader (Biochrom EZ Read 400 Microplate Reader, Cambridge, United Kingdom).

**siRNA-DNA cotransfection.** HuH-7 cells were seeded at 7.5 × 10^4^ cells per well in a 24-well culture plate. At a confluence of 60–70%, cells were transfected with a mixture of anti-PTB siRNAs (25 nM of each, 75 nM in total): siRNA PTBP1; 5′-AACUUCCAUCAUUCCAGAGAA-3′, siRNA PTBP2; 5′-GAGAGGAUCUGACGAACUA-3′, and SMART pool siGENOME PTBP1 siRNA (number M-003528-02, GE Healthcare Dharmacon Inc., Piscataway, NJ, USA), as described in [46], together with the dl HCV-IRES (100 ng) and pSP64-Poly(A) plasmids using Lipofectamine 2000 system (#11668-019; Thermo Fisher Scientific, Waltham, MA, USA). As a control, 75 nM of Silencer Select Negative Control N°1 siRNA (#4404021, Thermo Fisher Scientific) was used (herein referred to as the scramble RNA, scRNA). After 24 h or 48 h of siRNA and dl HCV-IRES transfection, the cells were harvested, and the RLuc and FLuc activities were measured. RLuc RNA silencing was performed using 250 nM of *Renilla* siRNA (siRLuc) 5′-UAUAAGAACCAUUACCAGAUUUGCCUG-3′ (Integrated DNA Technologies, IDT, Coralville, IA, USA) using Lipofectamine 2000, as described in [55], together with dl HCV-IRES (200 ng), pcDNA3-1-LacZ (25 ng), and pSP64-Poly(A) (1 μg). When indicated, the His-PTBs expressing plasmid (1 μg) were added in place of the pSP64-Poly(A). As a negative control, 250 nM of scRNA was used. Then, 24 h after transfection, the culture medium was removed, the cells were harvested, and the RLuc and FLuc activities were measured.

**Western blotting.** Cells were lysed using the 1× of Passive Lysis buffer supplied with the DLR^TM^ Assay System (#E1960, Promega Corporation, Madison, WI, USA) 24 h post-transfection. The protein content was determined by a Bradford assay using the Bio-Rad protein assay (#500-0006, Bio-Rad Laboratories, Inc., Hercules, CA, USA). For recombinant PTB isoform detection, total protein (30 µg) was resolved on a 10% SDS/PAGE and transferred to a 0.45 µM nitrocellulose membrane (#10600002, Amersham, GE Healthcare, Life Sciences). For endogenous PTB detection, total protein (50 µg) was resolved on a 12% SDS/PAGE and transferred to a 0.45 µM nitrocellulose membrane. Membranes were blocked with TBS containing 5% skimmed milk and 0.1% Tween 20 for 1 h at room temperature, washed three times with TBS containing 0.1% Tween 20, and incubated overnight with the primary antibody. Monoclonal anti-polyhistidine antibody (#H1029, Sigma-Aldrich, St. Louis, MO, USA) at a 1:5000 dilution, anti-Flag antibody (#F3165, Sigma-Aldrich) at a 1:500 dilution, anti-GAPDH antibody (#MAS-15738, Thermo Scientific) at a 1:5000 dilution, or monoclonal anti-PTBP1 antibody (#8776S, Cell signaling Technology Inc., Danvers, MA, USA) at a 1:1000 dilution was used as primary antibody. An anti-mouse IgG-HRP conjugate (#074-1806; KPL Inc., Gaithersburg, MD, USA) or anti-rabbit IgG-HRP conjugate (#12-348, Sigma-Aldrich) was used as a secondary antibody at a 1:10,000 dilution. The expression of the recombinant protein was visualized by enhanced luminescence using a chemiluminescence reaction.

**Sequence and statistical analysis.** All the statistical data analysis and graphics were performed and made using the GraphPad Prism v9.0c program (La Jolla, CA, USA). BIOEDIT v7.0.9 (Ibis Biosciences, Carlsbad, CA, USA) and Vector NTI v11 (Invitrogen, Waltham, MA, USA) were used for sequence alignments and analysis. The sequences of all plasmids used in the study were confirmed (Psomagen, Inc., Brooklyn, NY, USA).

## 3. Results

### 3.1. Knockdown of Endogenous PTB Negatively Impacts HCV IRES Activity in HuH-7 Cells

**Figure 1:**
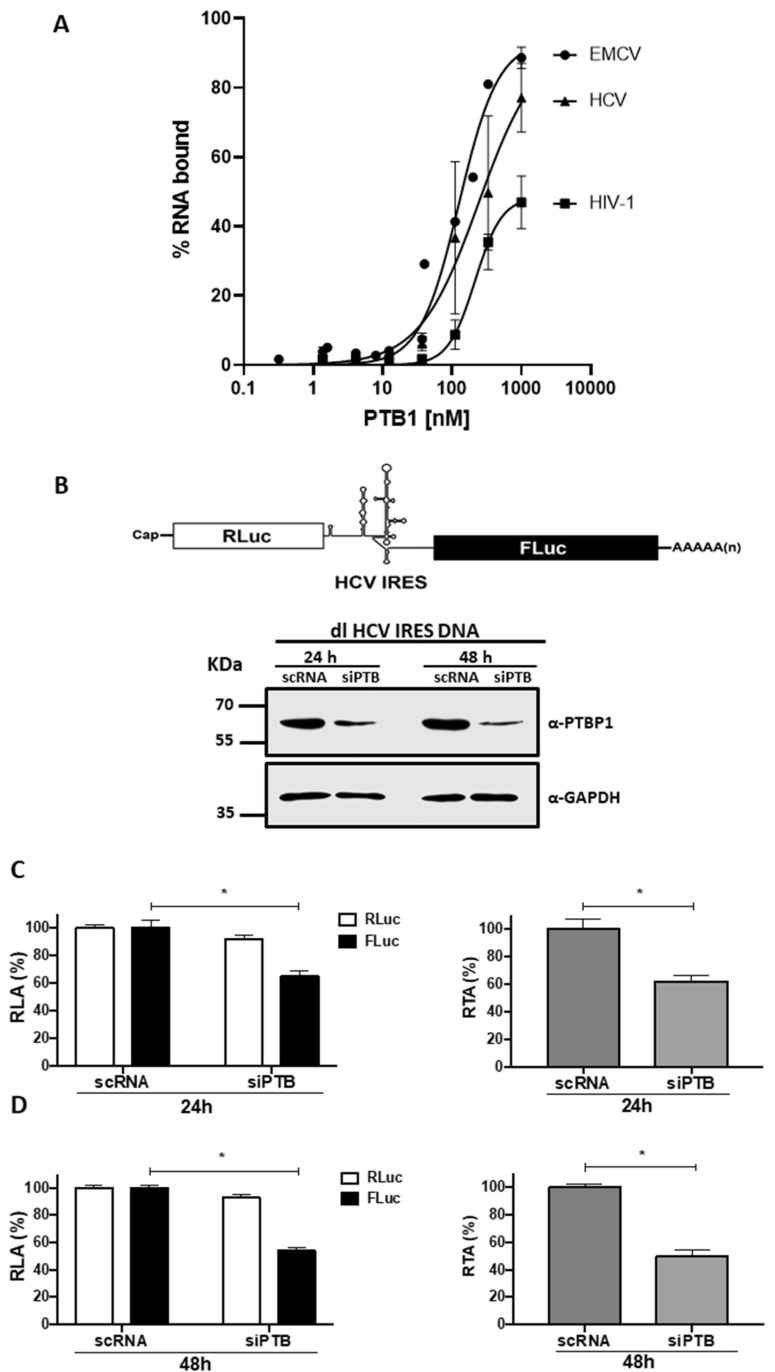
PTB1 binds to the 5′UTR of the HCV mRNA and modulates HCV IRES activity. (**A**) Binding curves of ^32^P-labeled in vitro synthesized HCV 5′UTR (▲), EMCV 5′UTR (•), or HIV-1 5′UTR (■) to purified His-PTB1 [38,56]. Data were fitted to the non-linear regression curve for specific binding with Hill slope. (**B**,**C**) HuH-7 cells were transfected with a scrambled (sc) RNA or a siRNA designed to target PTB mRNA(siPTB), together with the dl HCV IRES plasmid, as indicated in Material and Methods. (**B**) Upper panel: Schematic representation of the *Renilla* luciferase (RLuc) and firefly luciferase (FLuc) bicistronic constructs used in this study. Knockdown of PTB 24 and 48 h after transfection was confirmed by Western blotting (lower panel) using an anti-PTBP1 antibody. (**C**,**D**) RLuc and FLuc activities were measured 24 h (**C**) and 48 h (**D**) post-transfection and expressed relative to the scRNA control set to 100% (RLA: relative luciferase activity, left panels) or as relative translational activity (RTA), which corresponds to the [FLuc/RLuc] ratio (right panel). Values shown are the means (±SEM) of eight independent experiments, each conducted in duplicate. Statistical analysis was performed by the ANOVA test, followed by Sidak’s multiple comparisons tests; * *p* < 0.05 vs. the negative control.

The impact of PTB on HCV IRES activity remains controversial [18,19,20,21,23,24,25,26]. First, we sought to determine whether PTB1, the most characterized isoform, binds to the 5′UTR of the HCV mRNA. For this, a filter binding assay was conducted using a purified histidine-tagged (His)-PTB1 protein and a radiolabeled in vitro transcribed 452 nt long RNA corresponding to the entire HCV 5′UTR (from nt 13 of domain I to the *FLuc* coding region), recovered from the dl HCV IRES plasmid [47], following well-established protocols [56]. Radiolabeled RNA corresponding to the 5′UTR (nts 265 to 840) of the encephalomyocarditis virus (EMCV) RNA was used as a positive PTB1 binding control [46,57], and the 5′UTR of the genomic HIV-1 mRNA (nts 1 to 332) as a control for weak PTB1 binding [46,58,59]. In agreement with a previous report [46], PTB1 bound to the EMCV 5′UTR (Kd = 127 nM) (Figure 1A) and did not specifically interact with the HIV-1 5′UTR. Data also showed that in vitro PTB1 bound to the HCV 5′UTR with a lower affinity (Kd = 242 nM) than it did to the EMCV 5′UTR (Figure 1A).

Next, we wondered whether PTBs knockdown influenced HCV IRES activity in cells. We used the dl HCV IRES plasmid to study the IRES activity of the HCV 5′UTR in cells [47]. The dl HCV IRES plasmid encodes for a dual luciferase (dl) mRNA containing an upstream *Renilla* luciferase gene (*RLuc*), the HCV IRES, and a downstream firefly luciferase gene (*FLuc*) (Figure 1B) [47]. The use of this plasmid enables the study of the HCV IRES isolates from the rest of the viral mRNA, a strategy considered highly relevant, as PTB binds the HCV Core-ORF and the 3′UTR of the HCV mRNA, regulating HCV mRNA translation and viral replication [60,61,62,63,64]. As target cells, we selected to use the hepatocyte-derived cellular carcinoma cell line, HuH-7. The selection considered that HCV replicates predominantly within the hepatocytes, and others have shown that PTB modulates the HCV IRES activity in HuH-7 cells [20]. The dl HCV IRES plasmid was transfected in HuH-7 cells with a non-related scrambled control siRNA (scRNA) or with a cocktail of short interfering RNAs targeting the mRNAs of endogenous PTB isoforms (siPTB), as described in [46]. Treatment of cells with siPTB reduced the expression of endogenous PTB, monitored by Western blotting using a monoclonal anti-PTBP1 antibody for protein detection and GAPDH as a loading control (Figure 1B). The HCV IRES activity was followed using the FLuc activity as the readout, while the RLuc reporter gene served as an upstream cap-dependent translational control. Luciferase activities were measured at 24 h (Figure 1C) and 48 h (Figure 1D) post-transfection. Data were expressed as relative luciferase activity (RLA, left panel) with the RLuc and FLuc obtained from cells transfected with the scRNA set to 100%. At 24 h (~35% reduction) and 48 h (~46% reduction), a significant decrease in the HCV IRES activity (FLuc) was evidenced with no impact on cap-dependent translation (RLuc) in cells transfected with the siPTB RNAs (Figure 1C,D). As PTB knockdown did not significantly alter RLuc activity, the observed reduction in FLuc activity could not be attributed to reduced stability of the dl HCV IRES mRNA (Figure 1C,D, left panel). The decrease in HCV IRES activity is better illustrated when data are presented as relative translational activity (RTA, right panel), which corresponds to the FLuc/RLuc ratio, an index of IRES activity [46,47]. Reductions of ~38% and ~50% in RTA were evidenced at 24 and 48 h, respectively (Figure 1C,D, right panel). Thus, results indicate that PTB binds the HCV 5′UTR, and in agreement with a previous study that also used HuH-7 cells [20], we conclude that PTB contributes to HCV IRES-mediated translation.

### 3.2. Overexpression of PTB1 and PTB4, but Not PTB2, Promotes HCV IRES Activity

**Figure 2:**
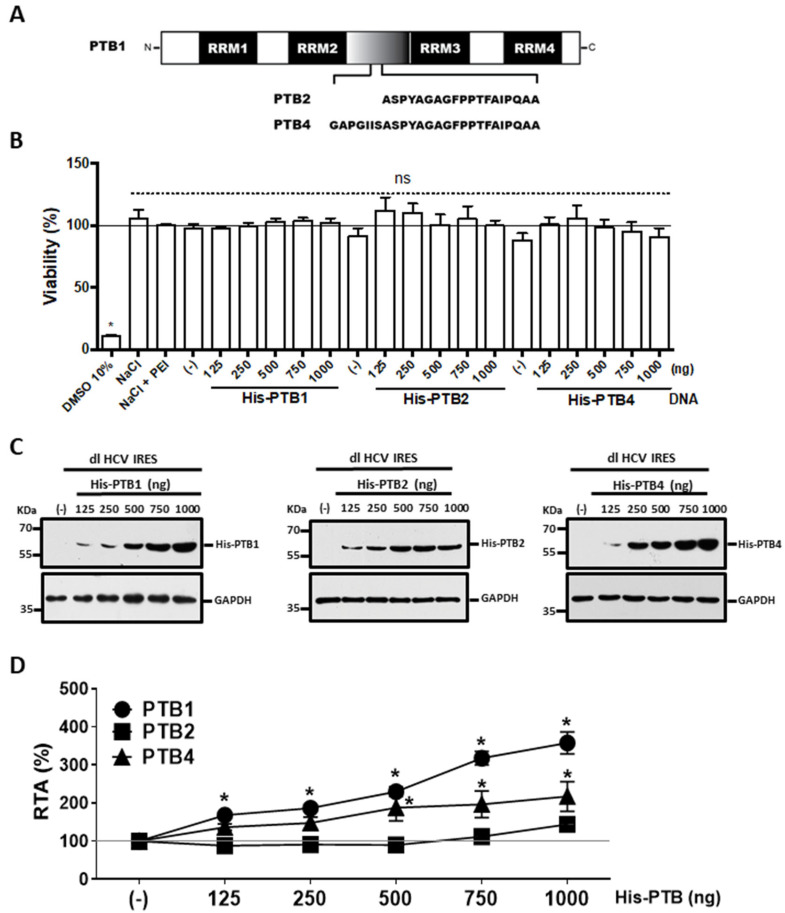
HCV IRES activity is stimulated in HuH-7 cells by PTB1 and PTB4, but not by PTB2. (**A**) Schematic representation of PTB isoforms showing the positions of the four RRMs. PTB2 and PTB4 differ from PTB1 by the insertion of 19 or 26 amino acids, respectively. (**B**,**D**) HuH-7 cells were transfected with the dl HCV IRES plasmid, the pcDNA3.1 LacZ control DNA (efficiency of transfection), and different concentrations (as indicated) of a vector expressing a His-tagged version of PTB1, PTB2, or PTB4. An empty pSP64-Poly(A) vector was used as the negative control (-). (**B**) The viability of HuH-7 cells was determined by measuring the cellular metabolic activity using the MTS assay. Dimethylsulfoxide (DMSO, 10%) was used as a control for cell death. (**C**) The expression of the His-tagged proteins was confirmed by Western blotting using an anti-His antibody (αHis). GAPDH was detected using an anti-GAPDH antibody (αGAPDH) as a loading control. (**D**) Luciferase activities were measured and expressed as RTA(%) relative to the activity measured in the HuH-7 cells transfected with dl HCV IRES, pcDNA3.1 LacZ, and pSP64-Poly(A) set to 100%. The values correspond to the means (±SEM) from three independent experiments each conducted in duplicate. Statistical analysis was undertaken by the ANOVA test, followed by Dunnett’s multiple comparisons tests; * *p* < 0.05 vs. the negative control; and ns, not significant.

PTB isoforms (Figure 2A), PTB1, PTB2, and PTB4, exert different effects on IRES activity [32,44,46,65]. Next, we investigated how PTB isoforms affect HCV IRES activity. For this, HuH-7 cells were cotransfected with the dl HCV IRES plasmid and pcDNA3-1-LacZ (25 ng), together with an irrelevant DNA (negative control, (-)), or increasing concentrations (125–1000 ng) of plasmids encoding for His-PTB1, His-PTB2, or His-PTB4. The overexpression of the PTBs did not impair HuH-7 cell viability (Figure 2B). The levels of β-galactosidase expression from plasmid pcDNA3-1-LacZ were quantified and used to determine transfection efficiency. Twenty-four hours post-transfection, protein lysates were obtained. The overexpression of the His-PTBs was confirmed by Western blot assays using an anti-His antibody and detecting GAPDH as a loading control (Figure 2C). Luciferase activities were measured, and data were expressed as RTA, with the values from cells transfected with the dl HCV IRES plasmid together with the control DNA (-) set to 100% (Figure 2D). Overexpression of His-PTB1 significantly stimulated HCV IRES activity (Figure 2D), reaching a maximum of a 3.5-fold increase when cells were transfected with the His-PTB1 (1000 ng) expressing plasmid. His-PTB4 overexpression also promoted HCV IRES activity with a maximum 2.1-fold increase at the highest used concentrations of the His-PTB4 expressing plasmid (1000 ng) (Figure 2D). The overexpression of His-PTB2 (125–1000 ng) did not significantly (1.4-fold increase) impact HCV IRES activity (Figure 2D). Based on these observations, we conclude that the overexpression of His-PTB1 and His-PTB4, but not His-PTB2, significantly stimulates the HCV IRES activity in HuH-7 cells.

### 3.3. Overexpression of PTB Isoforms Does Not Induce Alternative Splicing nor Cryptic Promoter Activity from the dl HCV IRES Reporter

**Figure 3:**
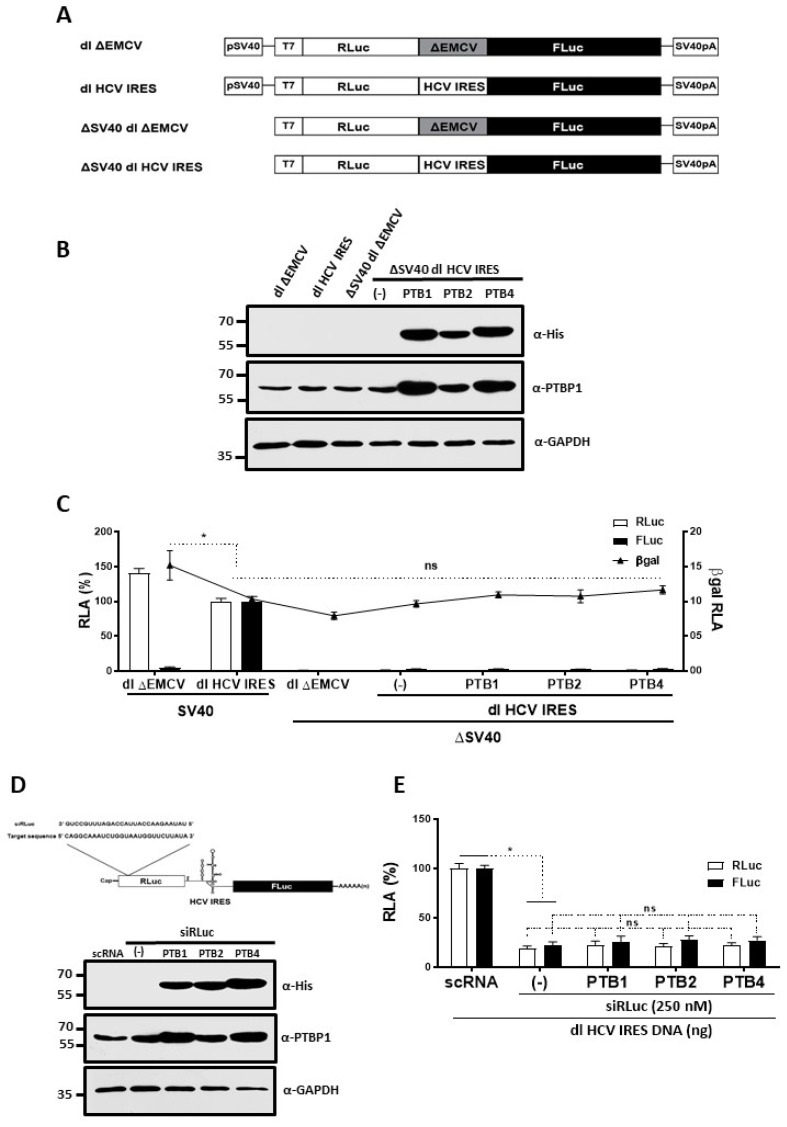
PTB isoforms do not induce alternative splicing nor cryptic promoter activity from the dl HCV IRES reporter. (**A**) Schematic representation of the *Renilla* luciferase (RLuc) and firefly luciferase (FLuc) bicistronic constructs. The SV40 and T7 promoters and the region corresponding to the defective encephalomyocarditis virus (∆EMCV) IRES are highlighted. The SV40 promoter from the dl HCV IRES was removed to generate the equivalent promoterless (∆SV40) vector. SV40pA represents the SV40 polyadenylation signal. (**B**,**C**) HuH-7 cells were transfected with 200 ng of the dl HCV IRES, dl ∆EMCV, ∆SV40 dl HCV IRES, or ∆ SV40 dl ∆EMCV DNA, the pcDNA3.1-LacZ DNA (25 ng), and the pSP64-Poly(A) empty vector (-) or plasmids expressing the PTB isoforms (1 μg). Total protein was extracted 24 h post-transfection. (**B**) Western blot detection of endogenous PTB (αPTBP1), recombinant His-PTB1, His-PTB2, His-PTB4 (αHis), and GAPDH (αGAPDH). (**C**) The RLuc and FLuc activities were measured, and activity obtained for the dl HCV IRES, pcDNA3.1-LacZ DNA, pSP64-Poly(A) mix was set to 100%. β-Galactosidase (β-Gal) expression was determined using the Beta-Glo Assay (Promega). Data are expressed as RLA with the β-Gal activity obtained in the mix dl HCV IRES/pcDNA3.1-LacZ/pSP64-Poly(A) set to 1. The values shown are the means (+/− SEM) for three independent experiments, each carried out in duplicate. (**D**,**E**) A scRNA (250 nM) control RNA or siRLuc (250 nM) RNA was cotransfected with the dl HCV IRES (100 ng), the pcDNA3.1-LacZ (25 ng), and the pSP64-Poly(A) vector (-) or plasmids expressing the PTB isoforms (1 μg). Total protein was extracted 24 h post-transfection. (**D**) Upper panel: Schematic representation of the siRLuc RNA strategy. Lower panel: Western blot detection of endogenous PTB (αPTBP1), recombinant His-PTB1, His-PTB2, His-PTB4 (αHis), and GAPDH (αGAPDH). (**E**) The RLuc and FLuc activities were measured and expressed relative to the values obtained when the dl HCV IRES/pcDNA3.1-LacZ/pSP64-Poly(A) mix was cotransfected with the scRNA, set to 100% (RLA: relative luciferase activity). The values in the figure correspond to the means of three independent experiments (±SEM). Statistical analysis was performed by the ANOVA test, followed by Dunnett’s multiple comparisons tests; * *p* < 0.05 vs. the negative control; and ns, not significant.

A previous study suggests that the dl HCV IRES plasmid might exhibit a cryptic promoter activity [66]. According to this report, in its DNA form, the HCV 5′UTR drives the expression of a downstream reporter gene by generating an RNA that specifically initiates within the HCV 5′UTR [66]. However, another study suggests that a single transcript is generated from bicistronic plasmids harboring the HCV 5′UTR in its intercistronic region [20]. If we presume that more than one transcript encoding for FLuc is generated from the bicistronic plasmid, the increase in FLuc activity (Figure 2D) would not necessarily reflect an increase in HCV IRES activity. Thus, we sought to determine if the FLuc activity from the dl HCV IRES plasmid could be credited to the presence of a cryptic promoter. In addition, we asked if the overexpression of the His-PTB isoforms promoted the expression of FLuc by stimulating any potential cryptic promoter activity in the transfected DNA [67]. For this, HuH-7 cells were transfected with the dl HCV IRES or the ΔSV40 dl HCV IRES, which lacks the eukaryotic simian virus 40 (SV40) promoter (Figure 3A). Plasmids were transfected in the presence or absence of the His-PTB isoform expressing plasmids (1000 ng). The dl ∆EMCV and ∆SV40 dl ∆EMCV plasmids were used as control reporters (Figure 3A). The dl ∆EMCV plasmid harbors a deleted 5′UTR of the EMCV mRNA, deficient in IRES activity [48], between the *RLuc* and *FLuc* ORFs (Figure 3A). To control for transfection efficiency, the pcDNA3-1-LacZ (25 ng) plasmid was added to the DNA mixtures. The expression of endogenous PTB, His-PTB1, His-PTB2, and His-PTB4 was monitored by Western blot (Figure 3B). Luciferase activity was measured, and results were expressed as RLA with the RLuc and FLuc obtained from cells transfected with the dl HCV IRES plasmid set to 100% (Figure 3C). RLuc activity was detected in cells transfected with dl HCV IRES and dl ∆EMCV (Figure 3C). Consistent with the lack of IRES activity in the dl ∆EMCV vector, FLuc activity was detected exclusively in cells transfected with the dl HCV IRES plasmid and not in those transfected with the dl ∆EMCV vector (Figure 3C). In lysates obtained from cells transfected with the ΔSV40 plasmids, neither RLuc nor FLuc activities were detected (Figure 3C). The β-gal activity in lysates from all transfected cells was independently measured and plotted relative to the activity obtained from cells transfected with the pcDNA3-1-LacZ, together with the dl HCV IRES plasmid, being set to 1 (Figure 3C). The results confirmed that the transfection efficiency, determined by β-gal activity, was similar in all the conditions, with the exception of the dl ∆EMCV vector, which transfected more efficiently (Figure 3C). The overexpression of His-PTB1, His-PTB2, or His-PTB4 did not stimulate FLuc expression from the ΔSV40 dl HCV IRES plasmid (Figure 3C). The results suggest that the dl HCV IRES plasmid does not exhibit cryptic promoter activity in HuH-7 cells even when in PTBs are overexpressed.

In cells, PTB regulates alternatively splicing of pre-mRNAs [29,32,33,39,68], raising the concern that FLuc expression could rise from a monocistronic mRNA generated by alternative splicing [67]. We wondered if the transcript generated from the dl HCV IRES plasmid was subjected to alternative splicing, which could be enhanced by PTBs overexpression. HuH-7 cells were transfected with the dl HCV IRES plasmid (200 ng) in the presence or absence of the His-PTBs plasmids (1000 ng) and a siRNA that targets the *Renilla* ORF (siRLuc) (250 nM) (Figure 3D, upper panel). As before, pcDNA3-1-LacZ (25 ng) plasmid was added to the mixture to control for transfection efficiency. Targeting the RLuc coding region is expected to knock down the bicistronic RNA without affecting the expression level of any potential monocistronic *FLuc* transcript [67]. The expression of endogenous PTB, His-PTB1, His-PTB2, and His-PTB4 in transfected cells was monitored by Western blot (Figure 3D, lower panel). In the presence of the siRLuc RNA, both RLuc and FLuc activities were significantly reduced, whether the His-PTB isoforms were overexpressed or not (Figure 3E). When directly compared, the reductions in RLuc and FLuc activities induced by the siRLuc RNA in the presence, or in the absence, of overexpressed His-PTB isoforms were not statistically different (Figure 3E). The results suggest that RLuc and FLuc expression levels were associated with a single transcript targeted by the siRLuc RNA. Therefore, in HuH-7 cells, the transcript generated from the dl HCV IRES plasmid is not subjected to alternative splicing. The results also show that the overexpression of the His-PTB isoforms does not induce any splicing event that would generate a monocistronic FLuc expressing mRNA from the dl HCV IRES RNA in HuH-7 cells. The results presented in Figure 3 validate the use of the dl HCV IRES plasmid and confirm that what was observed in Figure 2D corresponds to HCV IRES stimulation by PTB1 and PTB4.

### 3.4. PTB1 and PTB4 Hierarchy in Promoting HCV IRES-Mediated Initiation in HuH-7 Cells

**Figure 4:**
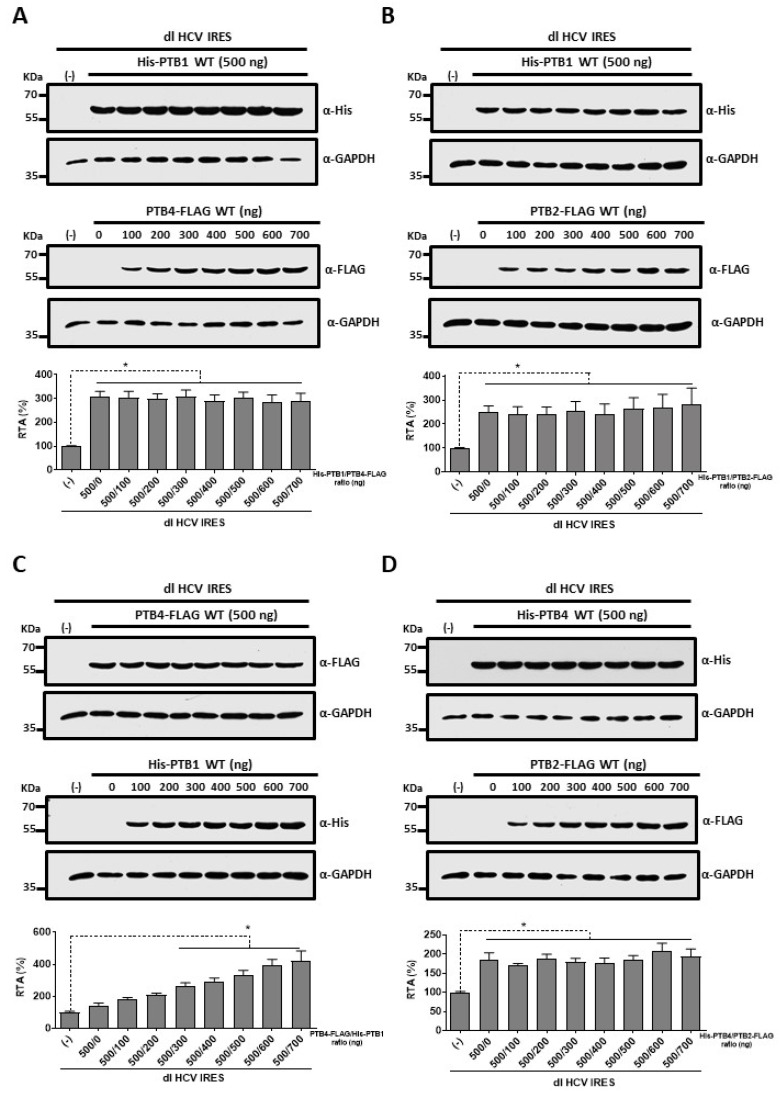
PTB1-induced stimulation of the HCV IRES activity is not altered by PTB4 or PTB2, but PTB1 complements PTB2. HuH-7 cells were cotransfected with the dl HCV IRES plasmid (100 ng), pcDNA3.1 LacZ DNA (25 ng), and PTB1 (500 ng) (**A**,**B**) or PTB4 (500 ng) (**C**,**D**) expressing plasmid, in combination with the indicated amounts of the plasmid expressing PTB4 (**A**), PTB2 (**B**,**D**), and PTB1 (**C**). The upper and middle panels show the detection of recombinant PTB isoforms and GAPDH by Western blotting. RLuc and FLuc activities were determined in the absence (pSP64-Poly(A)) or the presence of the indicated proteins, and data presented as RTA(%) are shown in the lower panel. The RTA value obtained in the absence of recombinant protein (-) was set to 100% (±SEM). Values are the means (±SEM) from three independent experiments each conducted in duplicate. Statistical analysis was performed by the ANOVA test, followed by Dunnet multiple comparisons tests; * *p* < 0.05 vs. the 500/0 point in each graph.

An earlier report from the laboratory suggests that the PTB1/PTB4, PTB1/PTB2, or PTB4/PTB2 ratios in cells regulate IRES-mediated translation initiation [46]. To determine if this was the case for the HCV IRES, we overexpressed combinations of the different PTB isoforms in HuH-7 cells. In the first series of experiments, a constant concentration of the His-PTB1 plasmid was transfected with an increasing concentration of the PTB4-FLAG (Figure 4A) or PTB2-FLAG (Figure 4B) expressing plasmid. The expression of His-PTB1, PTB4-FLAG, or PTB2-FLAG was confirmed by Western blot using antibodies for each tag. The levels of endogenous GAPDH protein were used as a loading control. Luciferase activities were measured and expressed as RTA. The mean RTA of the dl HCV IRES cotransfected with the control empty vector (-) set to 100% (±SEM) (Figure 4). PTB1 enhances HCV IRES activity. Co-expressing PTB4 (Figure 4A) or PTB2 (Figure 4B) had no additional effect on HCV IRES activity, suggesting that PTB4 or PTB2 cannot complement PTB1. Subsequently, the procedure was repeated using a constant concentration of the PTB4-FLAG or His-PTB4 plasmids and increasing concentrations of the His-PTB1 or PTB2-FLAG encoding vectors (Figure 4C,D). PTB4 alone increased HCV IRES activity, but IRES activity was further stimulated when PTB1 was added (Figure 4C). The addition of PTB2 to PTB4-expressing cells had no further effect on the activity of the HCV IRES (Figure 4D). These findings suggest that the hierarchy in promoting HCV IRES-mediated initiation in HuH-7 cells is PTB1 > PTB4 and confirm that PTB2 does not contribute to HCV IRES activity or the stimulation induced by either PTB1 or PTB4.

### 3.5. PTBs RRM1/RRM2 and RRM3/RRM4 Are Required for HCV IRES Stimulation in HuH-7 Cells

**Figure 5:**
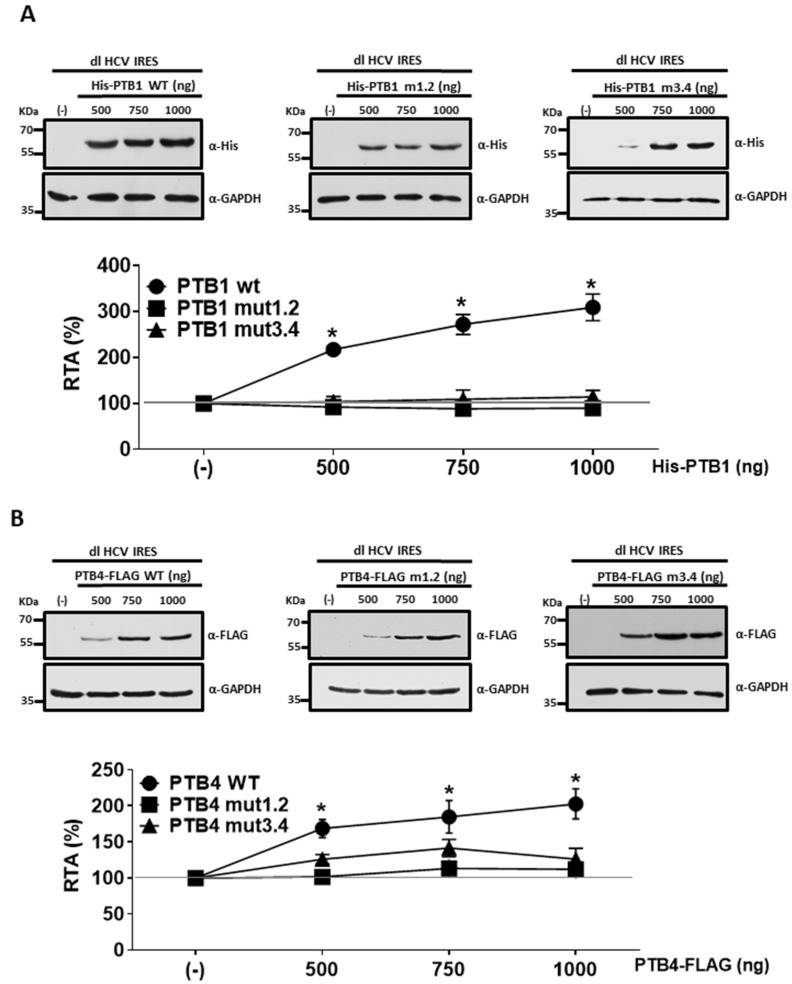
PTB1 and PTB4 require RRM1/RRM2 and RRM3/RRM4 to stimulate HCV IRES activity in HuH-7 cells. (**A**,**B**) HuH-7 cells were transfected with the dl HCV IRES plasmid (100 ng) together with the pcDNA3.1-LacZ (25 ng), and a vector expressing His-tagged versions of PTB1(wt), PTB1mut1.2, or PTB1mut3.4 or a FLAG-tagged version of PTB4(wt), PTB4mut1.2, or PTB4mut3.4. The pSP64-Poly(A) vector was used as a control (-). Expression of the tagged proteins was confirmed by Western blotting using an anti-His (αHis) (**A**) or anti-FLAG (αFLAG) (**B**) antibody. GAPDH was detected using an anti-GAPDH antibody (αGAPDH) as a loading control. Luciferase activities were measured and expressed as RTA(%) relative to the activity measured in the HuH-7 cells transfected with dl HCV IRES, pcDNA3.1-LacZ, and pSP64-Poly(A). Results for PTB1 and its mutants are shown in (**A**), and PTB4 and its mutants are shown in (**B**). Values are the means (±SEM) from three independent experiments. Statistical analysis was undertaken by the ANOVA test, followed by Dunnett’s multiple comparisons tests; * *p* < 0.05 vs. the negative control.

PTB binds the RNA to exert its function [29,39]. Next, we used mutants in RRM1/RRM2 and RRM3/RRM4 domains to understand how RRMs contributed to PTB1 and PTB4 ITAF function over the HCV IRES. PTB2 was excluded from the analysis, as its overexpression does not influence HCV IRES activity (Figure 2D). The rationale for studying RRM1/RRM2 and RRM3/RRM4 as clusters considered that PTB1 and PTB4 defer only in the linker domain between RRM2 and RRM3 (Figure 2A, and [29,32]) and that RRM3 and RRM4 act as a coordinated pair [39,40]. Thus, the dl HCV IRES plasmid was cotransfected in HuH-7 cells with an empty DNA plasmid (-) or plasmids expressing His-PTB1, His-PTB1mut1.2, His-PTB1mut3.4, PTB4-FLAG, PTB4mut1.2-FLAG, or PTB4mut3.4-FLAG. Mutations in the RRM domains in these plasmids have been described in [69], and they correspond to the m (RRM1), b (RRM2), f (RRM3), and k (RRM4) mutations, shown to disrupt the respective RRM’s ability to bind RNA [69]. As before, the presence of the recombinant proteins in transfected HuH-7 cells was confirmed by Western blotting using an anti-His antibody for the His-tagged PTB1s (Figure 5A, upper panel) or an anti-FLAG antibody for FLAG-tagged PTB4s (Figure 5B, upper panel). GAPDH was used as a loading control (Figure 4B and Figure 5A). The luciferase activities were measured, and data presented as RTA confirm that overexpression of His-PTB1 (Figure 5A) or PTB4-FLAG (Figure 5B) promotes HCV IRES activity in a concentration-dependent fashion. In these assays, a ~3-fold maximal stimulation was obtained with His-PTB1 (1000 ng of plasmid), and a maximum ~2-fold increase was obtained with PTB4 (750 ng of plasmid). Mutations in either RRM1/RRM2 or RRM3/RRM4 completely abrogated the stimulation of HCV IRES activity by PTB1 (Figure 5A) and PTB4 (Figure 5B). We conclude that the RNA binding activity of RRM1/RRM2 and RRM3/RRM4 is necessary for PTB1 and PTB4 to stimulate HCV IRES activity in HuH-7 cells.

### 3.6. Impacts of PTB1 and PTB4 RRM1/RRM2 and RRM3/RRM4 Mutations on HCV IRES Activity in HEK293T Cells

**Figure 6:**
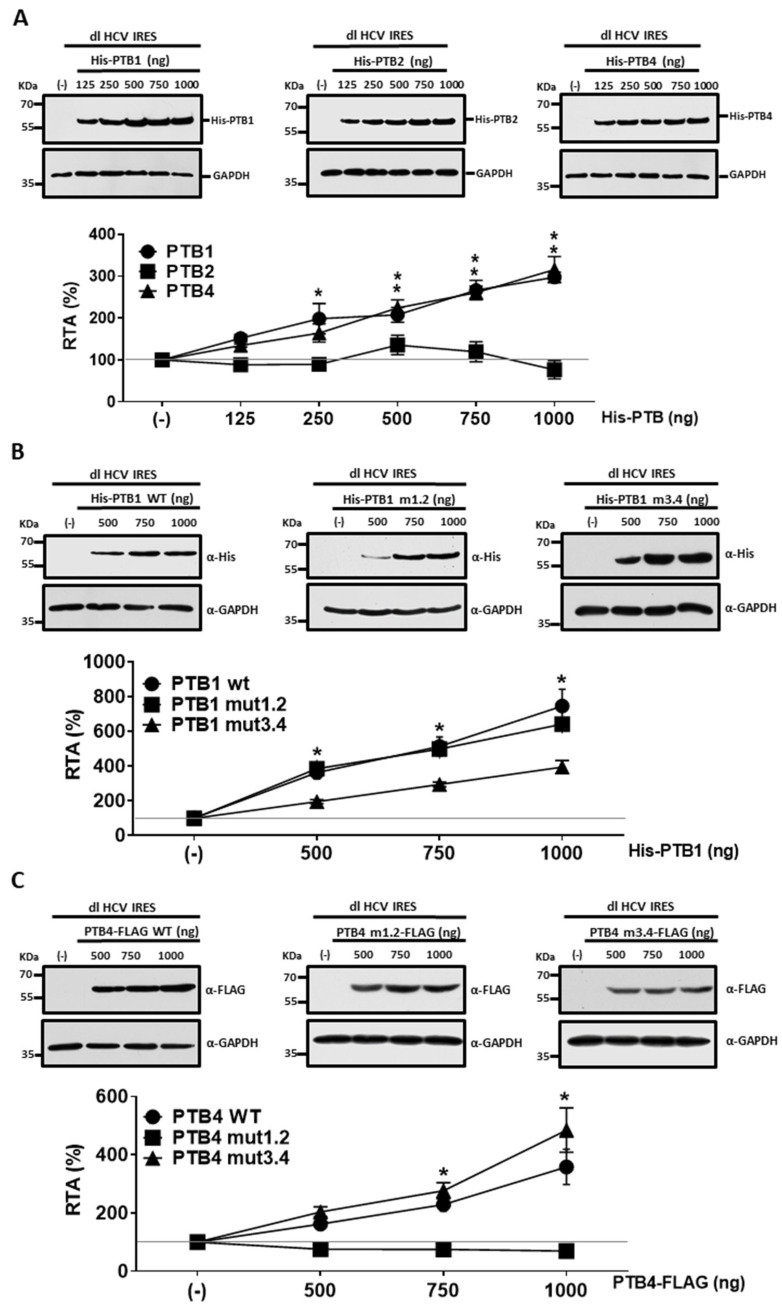
HCV IRES activity is stimulated in HEK293T cells by PTB1 and PTB4. (**A**) HEK293T cells were transfected with the dl HCV IRES plasmid (100 ng), pcDNA3.1 LacZ (25 ng), and different concentrations (as indicated) of a vector expressing a His-tagged version of PTB1, PTB2, or PTB4. The pSP64-Poly(A) vector (1 μg) was used as negative control(-). Expression of the tagged proteins was confirmed by Western blotting using an anti-His antibody (αHis). GAPDH was detected using an anti-GAPDH antibody (αGAPDH) as a loading control. Luciferase activities were measured and expressed as RTA(%) relative to the activity measured in the HEK293T cells transfected with dl HCV IRES, pcDNA3.1-LacZ, and pSP64-Poly(A). Values are the means (±SEM) from three independent experiments. Statistical analysis was undertaken by the ANOVA test, followed by Dunnett’s multiple comparisons tests; * *p* < 0.05 vs. the negative control. (**B**,**C**) HEK293T cells were transfected with the dl HCV IRES plasmid (100 ng), pcDNA3.1-LacZ (25 ng), and a vector expressing His-tagged versions of PTB1(wt), PTB1mut1.2, or PTB1mut3.4 or a FLAG-tagged version of PTB4(wt), PTB4mut1.2, or PTB4mut3.4. Expression of the tagged proteins was confirmed by Western blotting using an anti-His (αHis) (**B**) or anti-FLAG (αFLAG) (**C**) antibody. GAPDH was detected using an anti-GAPDH antibody (αGAPDH) as a loading control. Luciferase activities were measured and expressed as RTA(%) relative to the activity measured in the HEK293T cells transfected with dl HCV IRES, pcDNA3.1-LacZ, and pSP64-Poly(A). Data obtained with PTB1 and its mutants are shown in (**B**), and results obtained with PTB4, and its mutants are shown in (**C**). Values are the means (±SEM) from three independent experiments. Statistical analysis was undertaken by the ANOVA test, followed by Dunnett’s multiple comparisons tests; * *p* < 0.05 vs. the negative control. When the difference between two overlapping points is significant with respect to their controls: (*) are presented one over the other.

PTBs are expressed in different tissues [35], yet HCV replication is restricted mainly to the liver and some extrahepatic compartments, such as peripheral blood mononuclear cells [70]. Thus, we wondered if our findings could be replicated in a non-hepatic and non-lymphoid cell line such as HEK293T cells. HEK293T cells are not permissive to HCV infection [71], yet are supportive of HCV IRES function [13,46,72,73]. Furthermore, a previous report from our laboratory shows that the overexpression of PTB1 enhances HCV IRES activity in HEK293T cells [46]. Hence, the experiments described in Figure 2 and Figure 5 were repeated in HEK293T cells (Figure 6). For this, HEK293T cells were cotransfected with the dl HCV IRES plasmid and pcDNA3-1-LacZ (25 ng), together with an irrelevant DNA (negative control, (-)), or increasing concentrations (125–1000 ng) of plasmids encoding for His-PTB1, His-PTB2, or His-PTB4. The overexpression of His-tagged PTBs was confirmed by Western blot assays using GAPDH as a loading control (Figure 6A, upper panel). Luciferase activities were measured, and data were expressed as RTA, with the values from cells transfected with the dl HCV IRES plasmid and the control DNA (-) set to 100% (Figure 6A). The overexpression of His-PTB1 and His-PTB4, but not His-PTB2, stimulated HCV IRES activity in HEK293T cells (Figure 6A). However, in contrast to what was observed in HuH-7 cells (Figure 2D), in HEK293T, His-PTB1 and His-PTB4 promoted HCV IRES activity to an equivalent extent (Figure 6A).

Next, the dl HCV IRES plasmid was cotransfected into HEK293T cells with pcDNA3-1-LacZ (25 ng); and an empty DNA plasmid (-) or plasmids expressing His-PTB1, His-PTB1mut1.2, His-PTB1mut3.4, PTB4-FLAG, PTB4mut1.2-FLAG, the PTB4mut3.4-FLAG (Figure 6B,C). The overexpression of the recombinant proteins in transfected HEK293T cells was confirmed by Western blotting (Figure 6B,C, upper panels). The luciferase activities were measured, and data presented as RTA confirm that the overexpression of His-PTB1 (Figure 6B) or PTB4-FLAG (Figure 6C) stimulates HCV IRES activity in a concentration-dependent manner. PTB1 mutations in RRM1/RRM2 did not impact the protein’s ability to stimulate HCV IRES activity (Figure 6B), in contrast to what was observed in HuH-7 cells (Figure 5A). Mutations in PTB1 RRM3/RRM4 domains showed a nonsignificant trend towards higher HCV IRES activity (Figure 6B). These data suggest that in the context of PTB1, RRM3/RRM4 is mainly responsible for the stimulation of HCV IRES activity in HEK293T cells. Mutations in PTB4 RRM1/RRM2 abrogated the protein’s ability to stimulate HCV IRES (Figure 6C). Unexpectedly, RRM3/RRM4 mutated PTB4 significantly enhanced HCV IRES activity to levels equivalent to the non-mutated PTB4. These data suggest that in the context of PTB4, RRM1/RRM2 is responsible for stimulating HCV IRES activity in HEK293T cells.

## 4. Discussion

PTB is a well-known ITAF for cellular and viral IRESs [29,74]. However, the requirement for PTB varies among different viral IRESs. PTB is an absolute requirement for the rhinovirus IRES [75]. In contrast, PTB is stimulatory rather than essential for the function of the EMCV IRES [76]. The Theiler’s murine encephalomyelitis virus (TMEV) IRES is marginally dependent on PTB [76]. In previous experiments, while evaluating the ITAF function of PTB in the mouse mammary tumor virus (MMTV) IRES, we found that PTB1 stimulated the activity of the HCV IRES in HEK293T cells [46]. This finding prompted us to further characterize the function of PTB as an ITAF for the HCV IRES. First, we confirmed that PTB1 binds the 5′UTR of the HCV mRNA, albeit binding to the HCV 5′UTR was weaker than to the EMCV mRNA 5′UTR (Figure 1). However, consistent with a role in translation, knockdown of PTB in HuH-7 cells reduced but did not abrogate HCV IRES activity (Figure 1). Thus, results suggest that PTB plays a stimulatory rather than essential role for HCV IRES activity (Figure 1). The overexpression of PTB1 and PTB4, but not PTB2, promoted HCV IRES activity in HuH-7 cells.

A previous report showed that a DNA bicistronic reporter harboring the HCV 5′UTR in the intercistronic region exhibited cryptic promoter activity [66]. Based on this report, we evaluated the validity of our experimental approach and our conclusions (Figure 3). Consistent with the findings of Gosert et al. [20], we show that the used bicistronic plasmid does not harbor cryptic promoter activity, nor is its transcript subject to alternative splicing (Figure 3). One possible explanation for the discrepancy between these studies is the order of the luciferases encoding genes within the bicistronic reporter plasmid. Herein and in Gosert et al. (2000), an RLuc-HCV 5′UTR-FLuc configuration was used, Dumas et al. (2003) used a FLuc-HCV 5′UTR-RLuc reporter. The FLuc reporter gene exhibits cryptic promoter activity [77]. However, the cryptic promoter lies within the *FLuc* coding sequence generating mRNAs that do not code for a functional luciferase enzyme [77]. Thus, when the FLuc ORF is positioned as the first cistron upstream of the intercistronic region, the cryptic promoter activity within the *FLuc* coding sequence might generate mRNAs expressing only the second cistron [66]. This was not evidenced when the *FLuc* coding sequence was positioned as the second cistron downstream from the intercistronic region (Figure 3 and [20]). These results indicate that what was observed in Figure 2D corresponds to genuine HCV IRES activity. Additionally, these findings corroborate that the overexpression of PTB1 and PTB4, but not PTB2, stimulated HCV IRES activity (Figure 2D). This result suggests that the different PTB isoforms play distinct roles in HCV IRES-mediated translation initiation (Figure 2D and Figure 4). The hierarchy of efficiency in promoting HCV IRES activity in the bicistronic mRNA assay was PTB1 > PTB4 in HuH-7 cells (Figure 2D and Figure 4). Thus, our findings confirm previous reports showing that PTB1, PTB2, and PTB4 impact biological processes, such as pre-mRNA splicing and IRES-mediated translation initiation, differently [32,44,45,46]. Our observations also agree with other reports showing that PTB isoforms differentially impact viral IRESs. For example, PTB1, PTB2, and PTB4, in that order, stimulate the human rhinovirus-2 (HRV-2) IRES [32]. PTB1 and PTB4, in that order, stimulate the MMTV IRES [46]. Interestingly, PTB2 is a negative modulator for the MMTV IRES [46]. However, PTB2 had no impact on the activity of the HCV IRES (Figure 3). The results also suggest that the ratio between the different PTB isoforms modulates HCV IRES activity in HuH-7 cells (Figure 4). PTB1 complemented the function of PTB4 (Figure 4C) yet PTB4 did not complement PTB1 in HuH-7 cells (Figure 4A). Adding further complexity to the regulation of the HCV IRES by the PTB isoforms, data showed that the ITAF function of PTB1 and PTB4 was cell-type dependent (Figure 2, Figure 5 and Figure 6). Noteworthily, HCV IRES is documented to function differently in different cell lines, such as HuH-7 and HEK293 cells. For example, HCV IRES activity is highest in the G2/M phase of the cell cycle in HuH-7 cells [78], and it is lowest during the G2/M phase in HEK293 cells [79]. Herein, data show that in HEK293T cells, PTB1 and PTB4 stimulated HCV IRES equally (Figure 6A). At the same time, they confirm that in HEK293Tcells, the overexpression of PTB2 does not impact HCV IRES activity (Figure 6A).

Close examination of the data also suggests that the mechanisms used by PTB1 and PTB4 to stimulate HCV IRES activity in HuH-7 and HEK293T cells may differ. In HuH-7 cells, mutations in RRM1/RRM2 or RRM3/RRM4 abrogated PTB1 and PTB4’s capacity to promote HCV IRES activity. This observation suggests that the action of PTB1 and PTB4 requires bridging distantly located pyrimidine tracts within the HCV RNA and bringing them into proximity by forming an RNA loop [39,80]. Alternatively, the binding of PTB1 or PTB4 to distantly-located pyrimidine tracts within the HCV RNA might constrain the flexibility of RNA structure, favoring its IRES activity [44,57]. As PTB2 is longer than PTB1 and shorter than PTB4, it is possibly incorrectly positioned over the HCV 5′UTR, and thus cannot exert a structural impact on the HCV RNA. In HEK293T cells, PTB1 RRM1/RRM2 mutants stimulated HCV IRES activity as the PTB1wt (Figure 6). In contrast, the PTB1 RRM3/RRM4 mutant showed only reduced stimulating activity over the HCV IRES (Figure 6). This suggests that in HEK293T the RNA binding through RRM3/RRM4 is sufficient to enable PTB1 to exert its maximum stimulating effect over the HCV IRES. However, the binding of PTB1 to the target RNA through RRM1/RRM2 only partially stimulates HCV IRES activity (Figure 6). In contrast to PTB1, PTB4 RRM3/RRM4 mutant promotes HCV IRES activity slightly better than PTB4wt. However, mutations in RRM1/RRM2 abrogate PTB4’s ability to stimulate HCV IRES in HEK293T cells. These observations indicate that in the case of PTB4, RNA binding through RRM1/RRM2 is required to enable the protein to stimulate HCV IRES in HEK293T cells. The results in HEK293T are challenging to explain because PTB1 and PTB4 only vary by 26 amino acids present in the linker region between RRM1/RRM2 and RRM3/RRM4 (Figure 2A). Nevertheless, we can speculate that when compared to hepatic cells, in non-hepatic cells, PTB isoform concentration or ratios might differ [33,34,35]. Additionally, partner components and HCV IRES transacting factors, such as miR-122, vary in concentration between hepatocytes and other cell types [81,82,83]. The possible role of PTB partner proteins in regulating IRES activity is supported by studies showing that PTB/PCBP2 stimulates the PV IRES [75], and PTB/Unr/PCBP2 promotes the HRV IRES activity [84]. PTB/Ebp1 facilitates the binding of eIF4G/4A to the FMDV IRES [85]. Supposing that PTB is part of an RNA-binding protein (RBP) complex that works as a modulator of HCV IRES-mediated translation as for other IRESs [36,74,84,85,86], it is conceivable to propose that the composition of the protein complex might vary from one cell type to another. This component variation might alter the mechanism by which the complex, or any of its components, such as PTB, interacts with its target RNA. This suggests that remodeling the PTB-containing protein complex in different cell types or even under different physiological conditions could have dissimilar impacts on the HCV IRES activity [74,86]. This could partly explain the associations of PTB with IRES-dependent cellular activity, IRES associated-host tropism [65,75,84,87], and IRES-mediated virus attenuation [88]. Though interesting, our interpretation remains highly speculative. Further RNA–protein structural studies are required to understand PTB’s mechanisms of action of the HCV IRES. In conclusion, our results provide novel insights into the regulation of the IRES-activity of HCV mediated by the different isoforms of PTB, contributing to understanding the mechanism of viral-IRES-mediated translation initiation and its tight regulation by host proteins.

## Data Availability

Not applicable.
